# The Effect of Gender and Menstrual Phase on Serum Creatine Kinase Activity and Muscle Soreness Following Downhill Running

**DOI:** 10.3390/antiox6010016

**Published:** 2017-02-23

**Authors:** Tanja Oosthuyse, Andrew N. Bosch

**Affiliations:** 1 Exercise Laboratory, School of Physiology, University of the Witwatersrand, Medical School, Johannesburg 2193, South Africa; 2 Division for Exercise Science and Sports Medicine, Department of Human Biology, University of Cape Town, Cape Town 7700, South Africa; andrew.bosch@uct.ac.za

**Keywords:** oestrogen, progesterone, eccentric exercise, membrane stability, creatine kinase, delayed onset muscle soreness

## Abstract

Serum creatine kinase (CK) activity reflects muscle membrane disruption. Oestrogen has antioxidant and membrane stabilising properties, yet no study has compared the CK and muscle soreness (DOMS) response to unaccustomed exercise between genders when all menstrual phases are represented in women. Fifteen eumenorrhoeic women (early follicular, EF (*n* = 5); late follicular, LF (*n* = 5); mid-luteal, ML (*n* = 5) phase) and six men performed 20 min of downhill running (−10% gradient) at 9 km/h. Serum CK activity and visual analogue scale rating of perceived muscle soreness were measured before, immediately, 24-h, 48-h and 72-h after exercise. The 24-h peak CK response (relative to pre-exercise) was similar between women and men (mean change (95% confidence interval): 58.5 (25.2 to 91.7) IU/L; 68.8 (31.3 to 106.3) IU/L, respectively). However, serum CK activity was restored to pre-exercise levels quicker in women (regardless of menstrual phase) than men; after 48-h post exercise in women (16.3 (−4.4 to 37.0) IU/L; 56.3 (37.0 to 75.6) IU/L, respectively) but only after 72-h in men (14.9 (−14.8 to 44.6) IU/L). Parallel to the CK response, muscle soreness recovered by 72-h in men. Conversely, the women still reported muscle soreness at 72-h despite CK levels being restored by 48-h; delayed recovery of muscle soreness appeared mainly in EF and LF. The CK and DOMS response to downhill running is gender-specific. The CK response recovers quicker in women than men. The CK and DOMS response occur in concert in men but not in women. The DOMS response in women is prolonged and may be influenced by menstrual phase.

## 1. Introduction

Creatine kinase-MM (CK) is the muscle-specific isoform of the enzyme that catalyzes the reversible exchange of high-energy phosphate bonds between phosphocreatine and ADP to form ATP and creatine [1]. A portion of CK is bound to the M-line of the sarcomeres to facilitate ATP availability for myosin cross-bridge formation and contraction and is also present in other areas of the myofibre that have a high ATP demand [1]. Binding to the M-line is dependent on pH and therefore may be dependent on metabolic status [1]. Creatine kinase-MM appearance in serum can indicate the occurrence of both metabolic and mechanical disturbances within the sarcomere, while also reflecting plasma membrane integrity, stability and function [1,2].

Following eccentric exercise in men, peak serum CK activity has been reported to correlate with muscle soreness and decrements in maximal isometric strength [3], but nevertheless is often not regarded as a good measure to quantify exercise-induced muscle damage (EIMD) owing to high inter-individual variability. Serum CK activity does however provide an indication of plasma membrane function, which is a key element in the occurrence of EIMD [2] and accordingly, the CK response to exercise does not correlate with body mass, muscle mass, percent body fat or body mass index [3,4].

It is known that men have a higher resting serum CK activity than women [5] and eumenorrhoeic women have higher circulating oestrogen concentrations than men at most times during their menstrual cycle [6]. Oestrogen has known antioxidant properties [7] and membrane stabilising properties owing to oestrogen’s phenolic cholesterol-like structure, which allows it to intercalate within membrane phospholipids [2]. Animal studies have described a gender-effect associated with oestrogen’s action to reduce serum CK activity and histological evidence of muscle damage following exercise [8,9,10,11,12]. For this reason, it may be expected that oestrogen in women will protect against the leakage of CK following unaccustomed exercise such as downhill running that imposes eccentric contraction.

However, there is much contention whether a gender difference does in fact occur in the EIMD and CK response to exercise in humans [13,14,15]. Most studies have only evaluated a gender difference following maximum eccentric resistance-type exercise [4,16,17] and not following lower intensity aerobic eccentric or unaccustomed exercise. Furthermore, most gender studies have compared only absolute CK activity [4,16,17] and therefore failed to compare the CK response relative to pre-exercise values, which is necessary to isolate the effect of exercise over and above the established higher resting CK activity in men. One previous study that reported no difference between men and women did compare the CK response to downhill running expressed relative to pre-exercise values, but this study performed measurements only up to 24 h following exercise [18]. Another former study reports a greater serum CK activity in men than women following eccentric resistance exercise but measurements were only made following 4, 7 and 10 days of recovery, with earlier recovery time points omitted [4]. Furthermore, these studies [4,18] did not consider the menstrual phase or menstrual status of the women in their studies (i.e., eumenorrhoea, amenorrhoea, oral contraceptive use, postmenopausal, etc.) and others have included women in only one menstrual phase [16,17].

Resting serum CK activity has previously been reported to be higher in amenorrhoeic women athletes who have persistently low serum oestrogen concentrations compared with women athletes who have ovulatory menstrual cycles [19]. Women taking oral contraceptives (OC) tested during their active pill phase and therefore exposed to high exogenous synthetic oestrogen had a lower CK response 72 h after downhill running compared with eumenorrhoeic women not on oral contraceptives when tested in their mid-follicular phase with moderate to low endogenous oestrogen concentrations [20]. Similarly, postmenopausal women on hormone replacement therapy had lower serum CK activity following eccentric exercise than those not taking hormone replacement therapy [21]. Furthermore, a recent study found the serum CK response and decline in muscle strength after maximal eccentric quadriceps contractions to be greater in men and in women taking OC when tested during their placebo-pill phase compared with eumenorrhoeic women not taking OC also tested during their early follicular phase but with a modestly higher oestrogen concentration [16]. Therefore, the CK response to exercise appears to be lower when women are tested during periods coincident with higher oestrogen concentrations than when tested in conditions of lower oestrogen states.

However, progesterone may be a modulator of oestrogen in the luteal phase of the menstrual cycle. For example, in the condition of luteal phase-deficiency in women, which is characterised by a shortened luteal phase with suppressed progesterone secretions but normal oestrogen concentrations, resting CK activity is lower than in women who have a normal luteal phase characterised by concurrently elevated oestrogen and progesterone [19]. Furthermore, in a study in women (part taking OC and part naturally cycling), the luteal phase characterised by concurrently elevated oestrogen and progesterone, produced greater strength decrement and higher CK activity at 96 h after intensive eccentric resistance exercise than in the follicular phase with modest to low oestrogen and low progesterone [22]. However, oral contraceptive use increases basal inflammatory markers and oxidative stress [23,24] and alters the hormonal response to exercise compared with naturally cycling women [25]. Moreover, the potency of the exogenous synthetic oestrogen (ethinyl oestrogen) is far greater than endogenously secreted oestrogens and circulating levels of the synthetic oestrogen is not reflected in the routinely measured serum 17β-oestradiol concentration. Furthermore, the extended half life of the exogenous synthetic estrogens can persist for a few days into the placebo week, making it difficult to distinguish between the action of the measured endogenous hormone level and the likely remnants of active exogenous hormone [6]. Thus the inclusion of part OC users and part naturally cycling women in a sample group may complicate interpretation of data. Therefore, the influence of gender and menstrual phase on the CK and muscle soreness response to unaccustomed exercise is unclear. Gender studies including only women who are naturally cycling and therefore without OC use and where all menstrual phases are included is lacking. Furthermore, no previous study has considered the CK response during the late follicular (LF) or pre-ovulatory phase characterised by the highest spike in oestrogen concentration without an increase in progesterone.

While delayed onset muscle soreness (DOMS) following unaccustomed exercise may occur as a consequence of direct micro-trauma in myofibres, it can in fact occur in the absence of apparent microscopic damage and the pathways that lead to the activation of muscle nociceptors are still being discovered [26]. DOMS may arise because of the inflammation and associated oedema that ensues following unaccustomed exercise, which is partly promoted by a rapid intramyocellular influx of calcium owing to compromised sarcolemmal membrane function [2,27]. Accordingly, evidence exists for women compared with men or conditions of increased oestrogen concentration to moderate both the inflammatory [12,17] and muscle oedema [28] response to exercise, which therefore could be expected to reduce muscle soreness or DOMS. While some previous gender comparative studies have reported a reduced perception of muscle soreness following eccentric exercise in women than men [28,29], another study reported no gender difference, but interestingly, the CK and DOMS response to exercise correlated in the men but not in the women [4]. Perceived muscle soreness, however, was not different in premenopausal women with differing ovarian hormone profiles in studies that included both women who use OCs and those with natural menstrual cycles [20,22] or in postmenopausal women taking hormone replacements and those not [21]. Although, in one study DOMS was less in OC-users during their active pill phase than in naturally cycling women during their mid-luetal, high ovarian hormone, phase [30], and indicates a possible difference in the perception of muscle soreness between OC and non-OC users. Thus, differences in DOMS have not been adequately investigated in men and women with natural menstrual cycles where all menstrual phases are considered.

This study aimed to determine whether the serum CK response (as an indication of plasma membrane function) and perception of DOMS to a bout of downhill running is influenced firstly by gender, where the timing of testing is done selectively in the women so as to include an equal distribution of early follicular, late follicular and mid-luteal menstrual phases. Whether the timeline for the recovery of serum CK activity and perceived DOMS occurs coincidently in the respective gender groups was also assessed. Secondly, differences between menstrual phases were investigated in the sub-groups of women.

## 2. Materials and Methods

### 2.1. Participants

Fifteen eumenorrhoeic women and six men that were young, healthy and not taking medication participated in the study (Table 1). Participants were sedentary and therefore were not following a regular training programme and performed less than 2 h of exercise per week that involved only mandatory movements associated with everyday life with minimal eccentric contractions. All participants provided written consent to participate in the study after being informed of the purpose, procedures and risks of the study. The study protocol was approved by the Human Research Ethics Committee of the University of the Witwatersrand (M980539).

### 2.2. Menstrual Cycle Characterisation

All women reported that they had not taken any oral contraceptives for at least six months prior to the study. For one complete menstrual cycle before and for the duration of experimental testing, the women recorded their oral temperature using a digital thermometer (Vital sign VS-10, Soar Corporation, Nagano, Japan) on waking each morning and determined the day of ovulation using a home ovulation kit (Clearplan, Unipath Ltd., Bedford, UK) by testing their morning mid-stream urine sample starting three days before the expected day of ovulation. The timing and duration of the respective menstrual phases were determined for each participant based on these measurements [31]. The women were then randomly assigned to one of three groups to be tested in one of three menstrual phases, i.e., early follicular (EF, *n* = 5), late follicular (LF, *n* = 5) and mid-luteal (ML, *n* = 5) phase groups. Experimental testing was initiated between the second and fifth day of the menstrual cycle in the EF group, between the days corresponding to two days prior to ovulation until the day of ovulation in the LF group, and between four to eight days after ovulation in the ML group. Menstrual phase was confirmed by measurement of serum oestrogen and progesterone concentrations at rest before exercise on the first day of experimental testing (Table 1). The serum concentration ranges for the ovarian hormones defining each menstrual phase was: EF, 100–320 pmol/L and <3.2 nmol/L; LF, 560–1430 pmol/L and <5.5 nmol/L; ML, 320–770 pmol/L and >16 nmol/L for 17β-oestradiol and progesterone, respectively.

### 2.3. Downhill Running Experimental Testing

No strenuous exercise was permitted 48 h before or for the duration of experimental testing and participants refrained from alcohol, antioxidant supplements or medication for the duration of their involvement in the study. All participants performed the downhill running intervention between 1300 and 1400 h to control for circadian variation. Participants ran on a treadmill (Powerjog EG.30, J.F. Electronics, Birmingham, UK) for 20 min at 9 km/h at a −10% gradient. Variables that increase the magnitude of EIMD due to eccentric exercise include: a greater number of eccentric muscle contractions, contractions at longer muscle lengths and a higher speed of contraction [26]. The degree of eccentric loading in downhill running is created primarily by the magnitude of muscle lengthening imposed by the negative gradient of the slope [32] and for this reason the gradient was maintained constant for all participants. Furthermore, the speed was maintained constant for all participants because a faster running speed compared with a slower running speed during downhill running may increase the fall from height and muscle fibre lengthening, the number of eccentric contractions for a given fixed time and the contraction speed and thereby cause a disproportionate difference in eccentric loading between participants [26,32]. Furthermore, the metabolic strain during downhill running is less than during flat or uphill running and cannot be equated at a given speed [33,34]. Thus, maintaining a fixed negative slope and fixed speed imposes the most uniform eccentric load between individuals and has been similarly applied in previous downhill running studies [35,36,37].

Delayed onset muscle soreness (DOMS) was determined by marking a 100 mm visual analogue scale (VAS) bordered by the terms “no pain” and “the worst pain ever experienced”. Participants rated their perception of soreness on rising from a seated position by using a pen to make a mark on the line to indicate their feeling of muscle pain relative to those two extreme points before exercise (−20 min), immediately after exercise (0 min), 24 h, 48 h and 72 h after exercise. The mark was then measured as the distance from the left border of no pain. The use of a VAS to evaluate pain has been previously validated [38] and has been used in the assessment of DOMS [3,4,22]. A 5 mL blood sample was taken at rest (−20 min), immediately after exercise (0 min), 24 h, 48 h and 72 h after exercise. Blood samples were collected in vacutainer tubes containing no additives and allowed to clot before centrifugation. Serum samples were stored at −20 °C until analysis. Resting serum 17β-oestradiol and progesterone concentration were measured by radioimmunoassay (Coat-A-Count, Diagnostic Products Corporation, Los Angeles, CA, USA) and serum creatine kinase-MM (CK) activity (EC 2.7.3.2) was measured in all samples using a spectrophotometric assay (Boehringer Mannheim) with an inter assay coefficient of variation of 2.6% and reliability of which has been validated against the International Federation of Clinical Chemistry reference method [39].

### 2.4. Statistical Analyses

Serum CK data was analysed by two-way ANOVA with repeated measures for a time effect. A Dunnett’s multiple comparison test was used to investigate a significant time effect relative to pre-exercise CK concentrations (−20 min). Differences in the absolute CK concentration between groups at given time points were investigated by Bonferroni’s multiple comparison test and described as means and standard deviation (SD). The magnitude of the CK response post exercise was described as the change relative to pre-exercise (post exercise minus pre-exercise at each time point) and expressed as the mean change and 95% confidence interval of the change and compared between groups by a one way ANOVA and Bonferroni’s post hoc test. A sample size of 15 women and 6 men is sufficient to provide >80% statistical power to detect a difference between groups for the change in serum CK activity from pre to post exercise of 20 IU/L with the current within-individual SD of 9 IU/L as calculated in our sample groups using the -20 min and 0 min serum CK values. Furthermore, a sample size of 15 women and 6 men is sufficient to provide 80% statistical power to detect a difference in absolute serum CK activity of 50 IU/L between groups at any given time with the current between-individual SD of 35 IU/L. Thus the sample sizes included in the current study were sufficient to detect small worthwhile changes. Anthropometry and the log-transformed ovarian hormone concentrations were compared by one way ANOVA and Bonferroni’s post hoc test. Cohen’s effect size scores were derived as a further descriptor for the magnitude of effect and are interpreted as 0–0.2 is trivial; 0.2–0.6 is small; 0.6–1.2 is moderate; 1.2–2.0 is large; > 2.0 is very large [40]. Pearson’s linear regression was used to test for correlations between the serum CK response relative to pre-exercise values and oestrogen concentration, body mass or perceived muscle soreness. *p* < 0.05 was regarded as significant.

## 3. Results

### 3.1. Anthropometry and Ovarian Hormone Concentrations

The men were taller and had greater body mass than the women but there was no difference in body mass index (BMI) (Table 1). Serum 17β-oestradiol and progesterone concentrations confirmed the correct timing of testing with the respective menstrual phases (Table 1). Serum 17β-oestradiol concentration was higher in the LF group than all other groups and higher in ML group than EF and men. Serum progesterone concentration was significantly greater in the ML group than all other groups (*p* < 0.0001). Serum 17β-oestradiol and progesterone concentration was not significantly different between the men and EF group.

### 3.2. Serum CK Response to Downhill Running

#### 3.2.1. Gender Comparison

**Figure 1:**
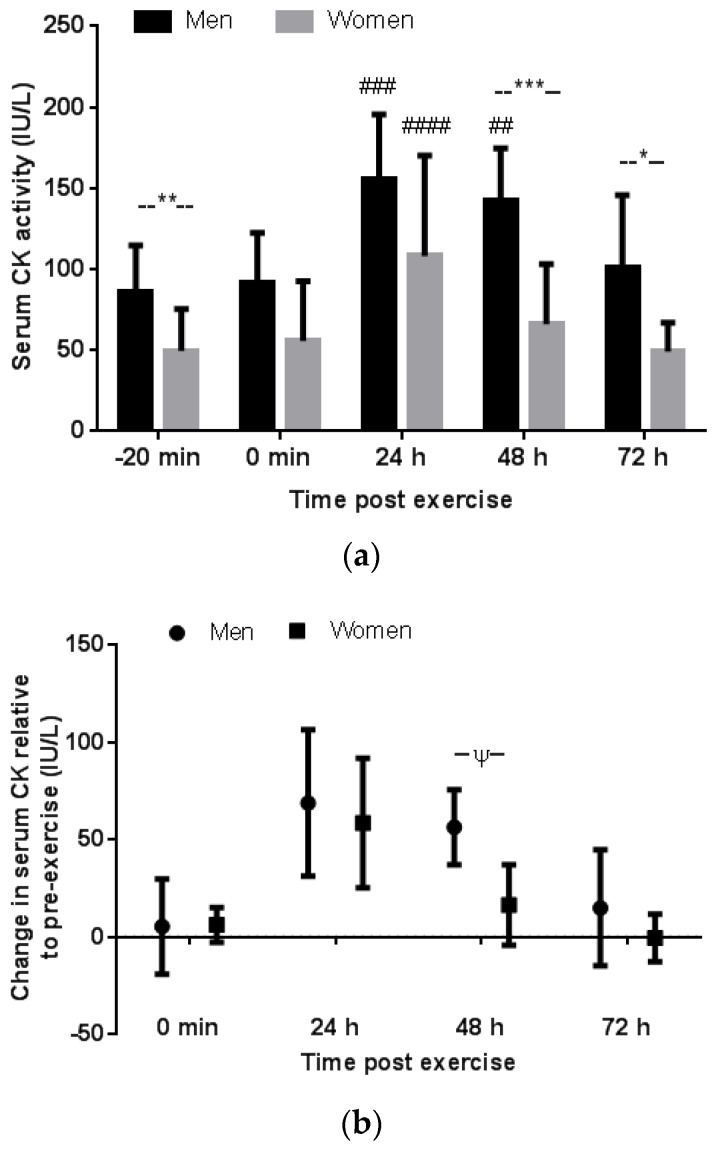
Gender comparison of the absolute serum creatine kinase (CK) activity pre- and post downhill running expressed as mean ± SD (**a**); and the magnitude of CK response post downhill running expressed as the average change relative to pre-exercise and 95% confidence interval (**b**). ##, ### and #### denotes a significant difference relative to pre-exercise (–20 min) within a group, *p* < 0.01, *p* < 0.001 and *p* < 0.0001, respectively. *, ** and *** denotes a significant difference between groups at that time point, *p* < 0.05, *p* < 0.01 and *p* < 0.001, respectively. ψ denotes a trend for difference between groups at this time point, *p* = 0.08.

Pre-exercise serum CK activity was greater in the men than women (mean difference (95%CI of the difference); 36.7 (10.1 to 63.3) IU/L, *p* = 0.01 and ES = 1.36) (Figure 1a). Serum CK activity increased above pre-exercise levels at 24 h post exercise in both the men (*p* = 0.0002, ES = 2.44) and women (*p* < 0.0001, ES = 2.28) with no difference in the magnitude of increase between groups (*p* > 0.99, ES = 0.22) (Figure 1b). Serum CK activity at 48 h post exercise had returned to pre-exercise values in the women (48 h verse pre-exercise: *p* = 0.31, ES = 0.64), but remained elevated above pre-exercise levels at 48 h in the men (*p* = 0.003, ES = 1.99). In the men, serum CK returned to pre-exercise levels only by 72 h post exercise (72 h verse pre-exercise: *p* = 0.76, ES = 0.53). The magnitude of increase in serum CK activity expressed as post exercise value relative to pre-exercise was similar between men and women at each time besides 48 h post exercise which tended to be greater in the men than women (*p* = 0.09, ES = 1.44) (Figure 1b). The absolute post exercise serum CK activity was greater in men than women at 48 h (*p* = 0.0003, ES = 2.22) and 72 h (*p* = 0.03, ES = 1.67) (Figure 1a). The 24-h post exercise serum CK response relative to pre-exercise did not correlate with body mass in men or women (*r* = 0.23, *p* = 0.65 and *r* = 0.037, *p* = 0.90, respectively).

#### 3.2.2. Menstrual Phase Comparison

**Figure 2:**
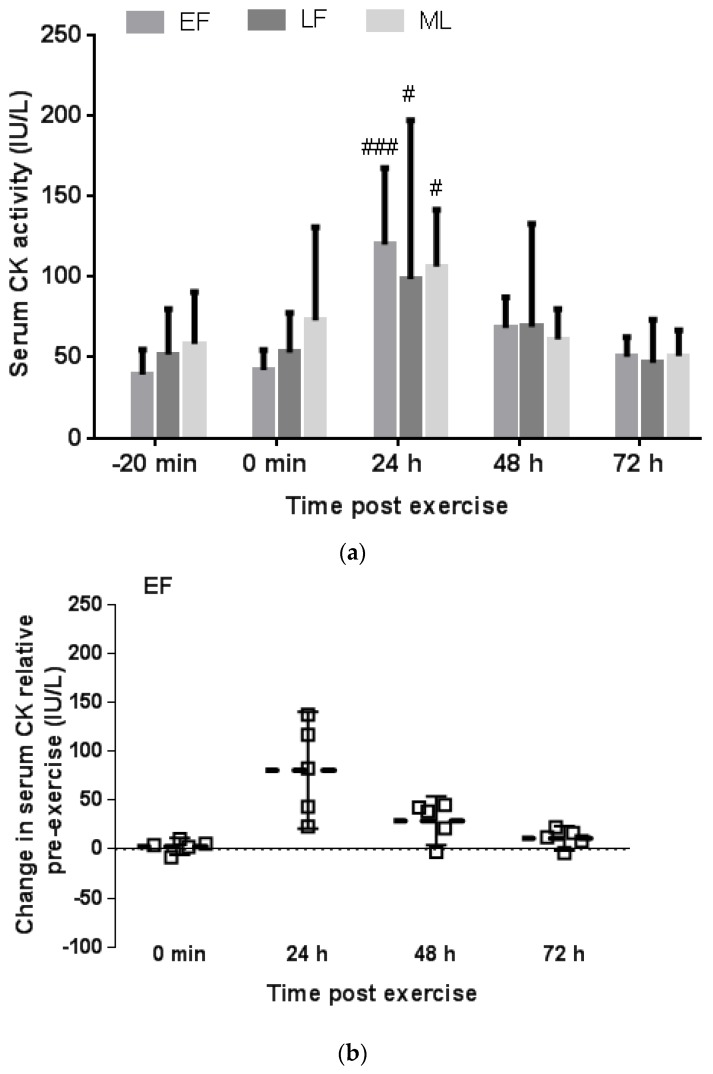

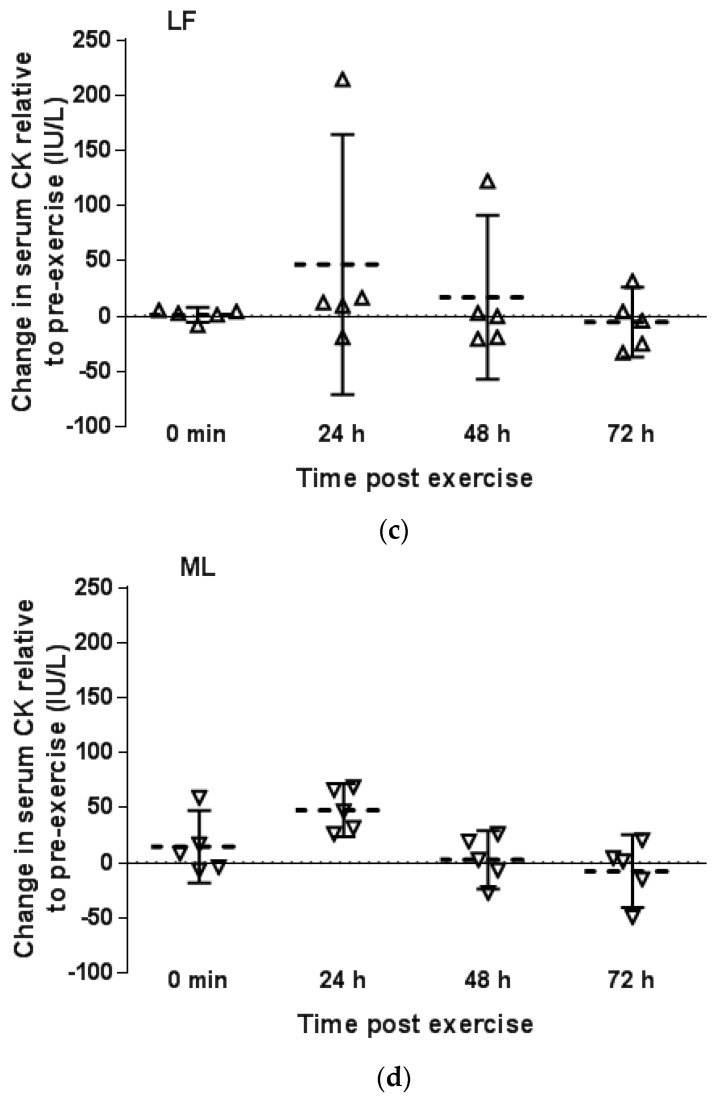
Menstrual phase comparison of the absolute serum creatine kinase (CK) activity pre- and post downhill running expressed as mean ± SD (**a**); and the magnitude of CK response post downhill running showing the individual data and average (---) change relative to pre-exercise and 95% confidence interval for early follicular (EF) group (**b**); late follicular (LF) group (**c**) and mid-luteal (ML) group (**d**). # and ### denotes a significant difference relative to pre-exercise (−20 min) within a group, *p* < 0.05 and *p* < 0.001, respectively.

**Figure 3:**
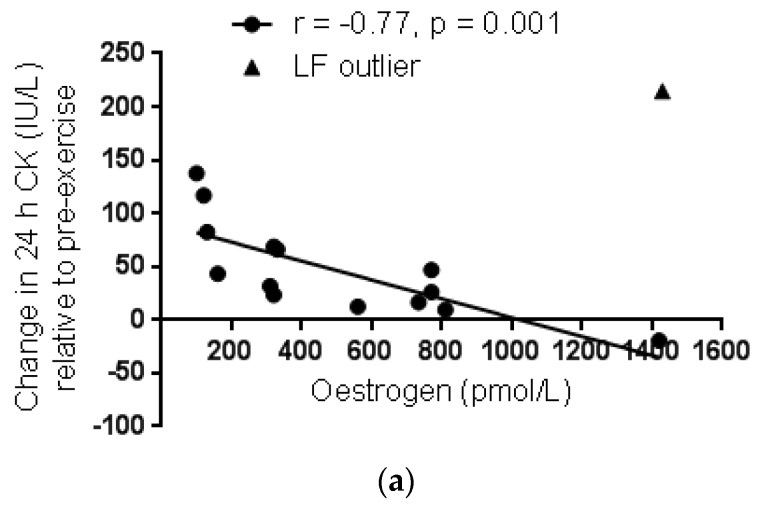

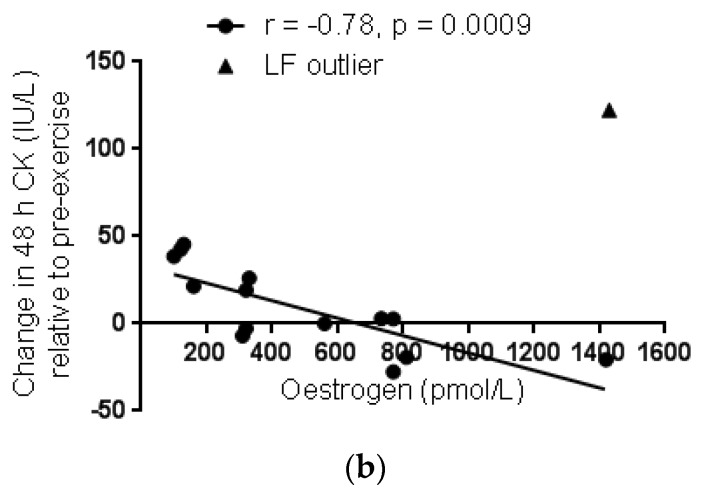
Linear regression of the change in serum creatine kinase (CK) activity over pre-exercise values at 24-h (**a**) and 48-h (**b**) post exercise with serum oestrogen concentration in women. Correlation analysis is excluding the one individual outlier from the LF group.

When the women were grouped according to menstrual phase, no significant difference was found in pre-exercise serum CK activity between menstrual phase groups (*p* = 0.52) (Figure 2a). Furthermore, the serum CK response to exercise peaked above pre-exercise values at 24 h in all menstrual phase groups (24 h verse pre-exercise; EF: *p* = 0.0003, ES = 3.15; LF: *p* = 0.048, ES = 1.83; ML: *p* = 0.042, ES = 1.87) and returned to pre-exercise levels by 48 h post exercise in all groups (48 h verse pre-exercise; EF: *p* = 0.34, ES = 1.13; LF: *p* = 0.76, ES = 0.67; ML: *p* = 0.99, ES = 0.11) (Figure 2b–d). Neither the 24-h nor 48-h CK response, expressed relative to pre-exercise, correlated with serum oestrogen concentration (*r* = −0.042, *p* = 0.88 and *r* = 0.037, *p* = 0.90, respectively). However, when data from an apparent outlier from the LF phase was omitted, a significant negative correlation was evident both for the 24-h and 48-h CK response expressed relative to pre-exercise and serum oestrogen concentration (*r* = −0.77, *p* = 0.001 and *r* = −0.78, *p* = 0.0009, respectively) (Figure 3a,b).

### 3.3. Perceived Muscle Soreness

#### 3.3.1. Gender Comparison

**Figure 4:**
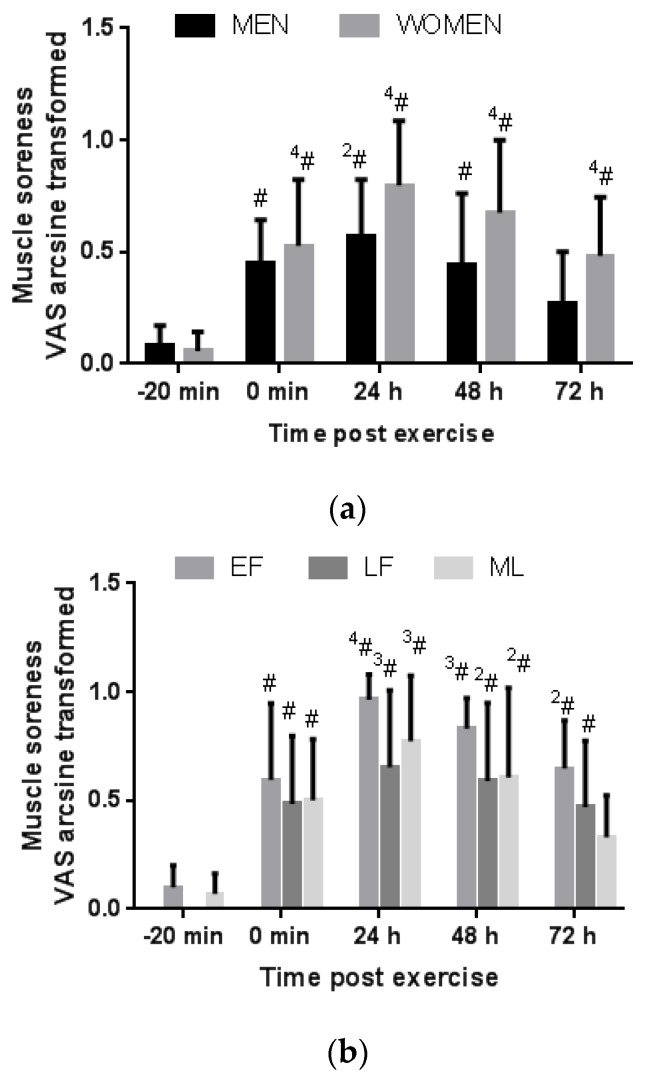
Comparison of visual analogue scale (VAS) rating of muscle soreness before and after downhill running between genders (**a**) and between menstrual phase groups (**b**). #, ^2^#, ^3^# and ^4^# denotes a significant difference relative to pre-exercise (-20 min) within a group, *p* < 0.05, *p* < 0.01, *p* < 0.001 and *p* < 0.0001, respectively.

There was no difference between the men and women in the VAS rating of perceived muscle soreness following downhill running at any time point (Figure 4a). In both groups VAS rating of muscle soreness increased above pre-exercise at 0 min, 24 h, and 48 h post exercise. By 72 h post exercise, the men reported no or low muscle soreness similar to pre-exercise (*p* = 0.57). Conversely, at 72 h post exercise, the women still reported elevated muscle soreness compared to pre-exercise (*p* < 0.0001) although less severe than at 24 h post exercise (*p* = 0.01). The 24-h and 48-h VAS rating did not correlate significantly with the 24-h and 48-h CK response relative to pre-exercise values in men (*r* = 0.64, *p* = 0.18 and *r* = 0.63, *p* = 0.18, respectively) or in women (*r* = 0.28, *p* = 0.31 and *r* = 0.41, *p* = 0.13, respectively).

#### 3.3.2. Menstrual Phase Comparison

When women were grouped according to menstrual phase, VAS rating of perceived muscle soreness was elevated above resting at all time points post exercise (0 min, 24 h, 48 h and 72 h) in the EF and LF phase groups (Figure 4b). In the ML group, VAS rating of muscle soreness was elevated above pre-exercise only at 0 min, 24 h and 48 h post exercise, but by 72 h post exercise scores were similar to pre-exercise ratings (*p* = 0.69). The 24-h, 48-h and 72-h VAS rating did not correlate significantly with serum oestrogen concentration in the women (*r* = −0.34, *p* = 0.21; *r* = −0.23, *p* = 0.41; and *r* = −0.12, *p* = 0.68, respectively).

## 4. Discussion

This is the first study to investigate a possible gender effect in the serum CK and DOMS response to unaccustomed exercise that included separate groups of women (all with natural eumenorrhoeic menstrual cycles) tested in three distinct menstrual phases. The novel findings of the current study are, firstly, that the magnitude of the 24-h peak CK response to downhill running is similar between men and women, however, circulating CK activity is restored to pre-exercise levels quicker in women than men; where CK activity is restored only after 72-h of recovery in men compared to 48-h post exercise in women regardless of menstrual phase. Secondly, while the initial perception of muscle soreness was similar between men and women, parallel to the CK response, the men report full recovery of muscle soreness symptoms by 72 h. Conversely, the women still report muscle soreness and incomplete recovery after 72 h despite CK levels being restored by 48 h. The delayed recovery of muscle soreness in the women appears mainly due to the women participating in their EF and LF menstrual phase and warrants further investigation.

The serum CK response to downhill running measured in the current study is of similar magnitude to that reported in a previous downhill running study [18]. The current study confirms the findings of this previous study of no gender difference in the 24-h post downhill running serum CK response. However, that former study did not perform further measurements at later recovery time points [18]. Contrary to the findings of the current study, Sewright et al. reported that men had a larger CK response to a bout of maximal eccentric resistance training than women [4]. However, the initial 4-day post measurement in this latter study [4] may have missed an earlier peak in CK activity that could have already recovered somewhat by day 4 in the women while still being elevated in the men. Similar to the findings of the current study, this latter study also found no difference in magnitude of soreness between sexes [4]. However, in that previous study, the men had a stronger relationship between the CK response, muscle soreness and strength loss than the women where muscle soreness did not correlate significantly with serum CK activity [4]; which agrees with the findings of the current study for a temporal association of concurrent time to recovery in serum CK activity and muscle soreness in the men but not the women, albeit without a significant correlation. Furthermore, in that latter study, women had a greater percentage strength loss than men despite the lower CK response [4]. Our findings support these previous findings that the CK response in women following unaccustomed exercise may not reflect the magnitude of DOMS or possibly muscle damage as well as it does in men.

Interestingly, in a recent gender comparative study, the serum CK response to exercise was greater in men than women in their placebo week of OC, despite lower endogenous serum oestradiol concentrations in the women than men [16]. However, the timing of testing corresponded to day 2–6 of the OC placebo week and exogenous oestrogens may still have been elevated owing to a long half-life of synthetic oestrogens [6]. A lingering presence of the synthetic oestrogens would also explain the lower concentration of endogenous serum oestradiol reported in the OC group compared to naturally cycling women group also tested on day 2–6 of their EF phase [16]. However, the lower CK response in the EF of the naturally cycling women compared to the men in that former study [16] confirm the finding in the current study for women in EF phase (with transiently low oestrogen concentrations similar to men), to still display a protective effect of a faster recovery of CK after unaccustomed exercise. Thus, it may be that certain effects of oestrogen, such as possible genomic effects [2], which are active during menstrual phases characterised by elevated oestrogen concentrations persist during the EF phase in women.

The quicker time to recovery of serum CK activity after downhill running in the women of the current study may be attributed to previous suggestions that oestrogen reduces the secondary phase of EIMD caused by local inflammation. This may be related to oestrogen’s membrane stabilising affect that reduces the intracellular calcium influx associated with EIMD, which promotes calpain protease activity leading to increased chemattractants and neutrophil infiltration [2,12]. This is supported by animal studies that found a reduced post exercise inflammatory response in female rats and male rats with oestrogen supplements compared to male rats without oestrogen supplements [41]. Furthermore, a previous gender study reported a reduced neutrophil inflammatory response in women in their ML phase compared with men after eccentric exercise [17]. Although a quicker recovery of serum CK activity may also suggest a greater rate of CK clearance in women, an in vitro study suggests that the gender difference is more likely owing to a decreased rate of CK leakage as it was shown that myofibrils in the presence of oestrogen resulted in a reduction in CK leakage [9]. Further animal studies suggest that oestrogen promotes faster muscle regeneration by increasing satellite cell activation and propagation [42]. However, we did not measure inflammatory or regenerative markers and therefore any gender difference in the inflammatory or repair response in the current study is indeterminate. Nevertheless, the faster time to restore baseline serum CK activity in women in the current study does provide evidence for a quicker time to restore plasma membrane function that may also likely indicate reduced damaging activity of reactive oxygen species. A previous gender study reports greater super oxide dismutase activity and reduced marker of lipid peroxidation in women in their ML phase than men after eccentric exercise [29].

The current finding of no difference in the CK response between menstrual phases is reported tentatively, owing to the small sample size for each menstrual phase group. In particular, the scatter plot showing individual responses reveals that the CK response in the LF phase was minimal in 4 of the 5 participants with only one individual outlier driving a strong positive response. Therefore, further studies are necessary to determine whether the LF phase corresponding with the pre-ovulatory spike in oestrogen with suppressed progesterone may in fact confer greater protection compared to other menstrual phases. Furthermore, only when the data from that one outlier is removed, does a significant negative correlation exist in the combined women group between the 24-h and 48-h post exercise CK response expressed relative to pre-exercise and serum oestrogen concentration. Similarly, a previous study that included OC women in their active pill phase and naturally cycling eumenorrhoeic women in their mid-follicular phase reported a negative correlation between the CK response to downhill running and pre-exercise oestrogen concentration [20]. The coincident increase in progesterone with oestrogen in the ML phase may modulate the protective effect of oestrogen [19] and may explain why no noticeable difference was observed in the current study between the CK response in the EF and ML phases despite a significantly higher oestrogen concentration in the ML phase.

The novel finding of delayed recovery of muscle soreness to occur in the EF and LF group but not in the ML group after downhill running is interesting. Previous findings that reported no difference in muscle soreness following eccentric exercise between follicular and luteal phase may be confounded by the inclusion of part OC users and nonusers [22] or measurement in mid-follicular phase with rising oestrogen concentrations [20]. In support of the current findings, a previous study reports greater perception of pain and rating of perceived exertion during exercise in women during the EF phase compared with the LF or ML phase [43]. The influence of the menstrual cycle on pain perception is complicated and not clearly described [44]. In fact, oestrogen and progesterone receptors occur on central and peripheral nerves and have been reported to induce both pronociceptive and antinociceptive affects where the overall effect may be dependent on the receptor type and relative presence of oestrogen and progesterone [44]. Nevertheless, most evidence suggests the occurrence of reduced perception of clinical pain in the ML phase and heighted pain when oestrogen concentrations are low such as in EF phase or when oestrogen concentrations reduce rapidly [44]. Moreover, patients with rheumatoid arthritis report greater joint pain and stiffness during the LF phase [44].

Future studies should include measures of muscle function in recovery from unaccustomed exercise in men and women during the EF, LF and ML phase in order to characterise gender or menstrual phase differences in EIMD per se. While changes in serum CK activity following exercise may more specifically indicate changes in plasma membrane function, both serum CK activity and DOMS may be poor markers to quantify EIMD as the CK response has high inter-individual variability and DOMS is subjective.

## 5. Conclusions

The CK and DOMS response to downhill running is gender-specific. While the initial CK response is similar between men and women, the CK response recovers quicker in women than men; with a seemingly similar time to recovery in all menstrual phases. The CK and DOMS responses occur in concert in men but not in women following downhill running. The DOMS response in women is prolonged and may be influenced by menstrual phase with possible prolonged muscle pain sensitivity in certain menstrual phases, which requires verification.

## Figures and Tables

**Table 1:** Participant characteristics.

Parameter	Men (*n* = 6)	Women (*n* = 15)	EF (*n* = 5)	LF (*n* = 5)	ML (*n* = 5)
Age (years)	21.8 ± 0.9	21.7 ± 2.4	20.7 ± 0.5	23.3 ± 2.4	21.2 ± 4.4
Body mass (kg)	71.4 ± 10.5	58.0 ± 10.2 ^a^	55.6 ± 7.8	57.6 ± 4.9	60.8 ± 16.2
Height (cm)	179.2 ± 8.5	160.6 ± 5.5 ^a^	158.1 ± 5.6 ^a^	161.6 ± 3.1 ^a^	162.1 ± 7.2 ^a^
BMI (kg/m^2^)	22.1 ± 1.8	22.4 ± 3.4	22.5 ± 4.7	22.0 ± 1.2	22.8 ± 4.0
17β-Oestradiol (pmol/L)	156.7 ± 51.6	552.3 ± 435.7 ^a^	166.0 ±88.8	990.8 ± 406.6 ^a,b,c^	500.0 ± 246.6 ^a,b^
Progesterone (nmol/L)	2.7 ± 0.5	10.8 ± 12.0	2.3 ± 0.9	4.1 ± 1.5	25.9 ± 8.2 ^a,b,d^
Length of MC (days)		29.4 ± 4.5	29.2 ± 5.3	29.2 ± 5.8	29.8 ± 2.5
MC day of running			3.6 ± 1.1	14.4 ± 2.7	20.4 ± 2.2

EF, early follicular; LF, late follicular; ML, mid-luteal; BMI, body mass index; MC, menstrual cycle. ^a^ denotes a significant difference to Men, *p* < 0.01; ^b^ denotes a difference to EF, *p* < 0.01; ^c^ denotes a trend for difference to ML, *p* = 0.09; ^d^ denotes a difference to LF, *p* < 0.0001.
